# Prevalence of Methicillin-Resistant *Staphylococcus aureus* (MRSA) in Egyptian Water Buffaloes and Risk Factors for Subclinical Mastitis

**DOI:** 10.1155/tbed/8862271

**Published:** 2025-07-01

**Authors:** Abdelfattah Selim, Mohamed Marzok, Hattan S. Gattan, Abdelrahman M. Hereba

**Affiliations:** ^1^Department of Animal Medicine (Infectious Diseases), Faculty of Veterinary Medicine, Benha University, Toukh 13736, Egypt; ^2^Department of Clinical Sciences, College of Veterinary Medicine, King Faisal University, Al-Ahsa 31982, Saudi Arabia; ^3^Department of Medical Laboratory Sciences, Faculty of Applied Medical Sciences, King Abdulaziz University, Jeddah, Saudi Arabia; ^4^Special Infectious Agents Unit, King Fahad Medical Research Center, King AbdulAziz University, Jeddah, Saudi Arabia; ^5^Department of Microbiology, College of Veterinary Medicine, King Faisal University, Al-Ahsa 31982, Saudi Arabia

**Keywords:** antibiotic-resistant, Egypt, PCR, *Staphylococcus aureus*, subclinical mastitis, water buffaloes

## Abstract

*Staphylococcus aureus* (*S. aureus*) is one of the main causative agents of mastitis, which results in severe economic losses. In addition, methicillin-resistant *S. aureus* (MRSA) has been reported in dairy farms and in water buffaloes. The present study aimed to determine the prevalence of subclinical mastitis (SCM) in water buffaloes, associated risk factors for SCM, and prevalence of MRSA in positive milk samples for SCM. Milk samples (*n* = 385) from buffaloes were examined using the California mastitis test (CMT), and *S. aureus* was detected in positive milk samples using bacteriological and biochemical tests. In addition, MRSA was identified in positive *S. aureus* samples using PCR targeting the *mecA* gene. The results revealed that the prevalence of SCM among water buffaloes in the studied areas was 43.6%, and 61.9% (104/168) were identified as MRSA based on PCR targeting the *mecA* gene. In vitro antibiotic susceptibility testing found cefoxitin to be resistant and linezolid to be sensitive against MRSA isolates. In addition, the statistical analysis revealed that there was no significant association between the prevalence of SCM and locality or duration of lactation. The prevalence of SCM was strongly associated with age, parity, absence of teat dipping, hand cleaning of milker hands between milking, and in animals with a history of mastitis. Regular CMT can detect early SCM and improve udder sanitation and milking hygiene. In addition, continuous testing of antimicrobial drugs against MRSA isolates is necessary due to the importance of *S. aureus* in public health and the development of antibiotic resistance, such as methicillin.

## 1. Introduction

Buffaloes are integral to Egypt's agricultural economy, contributing to milk and meat production, agricultural labor, and providing valuable manure [1]. Their role is crucial in supporting the livelihoods of many rural farmers and in promoting sustainable agricultural practices. However, research studies on buffalo have received less attention than studies on cattle [2].

Infectious illnesses are a serious barrier to livestock development in Egypt, particularly among buffaloes [3, 4]. Mastitis is one of the most serious diseases among the several infectious diseases that affect bovines, including buffaloes. *Staphylococcus aureus* is the primary cause of mastitis in bovines, rapidly adapts to the mammary glands' environmental circumstances, and spreads across animals during milking [5, 6].

Infection with *S. aureus* can induce tissue damage and gangrenous mastitis. Gangrenous mastitis is characterized by blue to black quarters that slough off and is commonly associated with staphylococcal alpha toxin production [7, 8].

*Staphylococcus aureus*, particularly methicillin-resistant *S. aureus* (MRSA), is a major pathogen of both veterinary and public health concern due to its ability to acquire and disseminate antimicrobial resistance [9]. The resistance mechanism in MRSA is primarily mediated by the *mecA* gene, which encodes for an altered penicillin-binding protein (PBP2a) that has a low affinity for β-lactam antibiotics, rendering them ineffective. This resistance, coupled with the pathogen's ability to form biofilms and evade the host immune response, contributes to persistent infections, such as subclinical mastitis (SCM) [10, 11].

MRSA has emerged in cattle because of the widespread and uncontrolled use of antibiotics, especially penicillin groups, to treat cows. Methicillin resistance in *S. aureus* has an adverse effect on treating diseases in humans and animals [12–14].

*Staphylococcus aureus* can develop methicillin resistance by acquiring the staphylococcal cassette chromosome (SCC) *mecA* gene, which modifies the penicillin-binding protein (PBP2) and reduces affinity for all β-lactam drugs [15–17].

In comparison to Europe, the United States had a higher prevalence of MRSA [18]. Studies have shown that prevalence rates were less than 50% in Portugal, Italy, and Greece, and above 70% in Vietnam and South Korea. Studies carried out in Egypt revealed that *S. aureus* strains resistant to cefoxitin and penicillin, which were identified from SCM, were highly prevalent in bovines in the governorates of Dakahlia and Ismailia [19, 20].

This study aimed to investigate the prevalence of SCM and MRSA infections in water buffaloes, alongside identifying the potential risk factors contributing to the occurrence of SCM.

## 2. Materials and Methods

### 2.1. Ethical Statement

The study protocol followed the guidelines and was approved by the ethics committee of the Faculty of Veterinary Medicine, Benha University. In addition, all methods were performed in accordance with the relevant guidelines and regulation ethics committee of the Faculty of Veterinary Medicine, Benha University. This study was conducted according to ARRIVE guidelines.

### 2.2. Study Area

**Figure 1:**
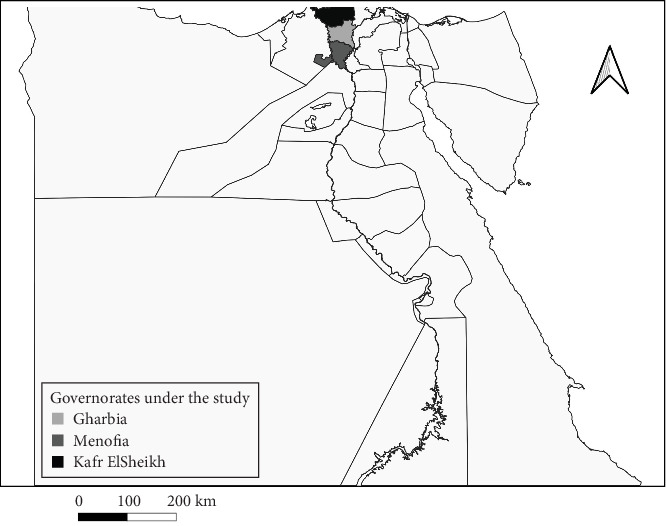
Map showed governorates under the study (map generated by QGis software).

The study was carried out in two governorates (Kafr El Sheikh and Menofia) located in Egypt's Nile Delta between January and December 2023. These governorates are situated geographically at 31°06′42′N 30°56′45′E and 30.52°N 30.99°E (Figure 1).

The selected governorates have a subtropical desert climate (classification: BWh) and are situated at an elevation of 30–33 feet above sea level. The annual temperature of the city is 25°C (74.8°F), and receives precipitation of 4 mm. These areas are a vital agricultural area and considered as a cornerstone of food production in Egypt.

### 2.3. Sampling and Sample Size

The sample size was calculated based on Thrusfield's formula [21]

  $$\begin{array}{c} n=Z^{2}P\left(1-P\right)/d^{2}, \end{array}$$

where *n* is the sample size, *Z* is the 95% confidence interval (CI), *P* is a predicted prevalence rate (50%), and absolute precision is 5%. A total of 385 buffaloes raised by individual farmers were analyzed; some of the animals displayed symptoms of mastitis, while others appeared normal (SCM).

The udder of the examined animal was washed using clean water, dried with clean tissue, and disinfected using 70% alcohol. After screening for SCM with the California mastitis test (CMT), milk samples were obtained [22]. Milk samples (8 mL) were collected from each animal in a clean, sterilized Falcon tube (15 mL) labeled with the number of the animal and the collection date.

A questionnaire was prepared in order to get animal data from farmers at the time of sampling. The questionnaire collected information on the animals, such as their age, parity, lactation stage, history of mastitis, presence of one or more lesions on the udders, and usage of antibiotics for mastitis.

### 2.4. Bacteriological Examination

The milk samples were thoroughly mixed and streaked on blood agar and incubated for 24 h at 37°C. In order to verify the presence of *S. aureus*, the bacterial colonies were streaked onto mannitol salt agar (MSA; Merck, Germany) and identified biochemically. *S. aureus* forms smooth, golden–yellow colonies with β-hemolysis on blood agar and ferments mannitol on MSA, turning the medium bright yellow. The suspected *S. aureus* colonies were confirmed microscopically by Gram staining and biochemically by coagulase and catalase tests [23].

### 2.5. Phenotypic Identification and Antibiotic Susceptibility Test for MRSA Strains

Fresh *S. aureus* colonies were adjusted to 0.5 McFarland and swabbed on Muller–Hinton agar. Oxacillin discs (1 μg) and cefoxitin (30 μg) discs were placed aseptically on Muller–Hinton agar (Oxoid, Sigma–Aldrich, UK). After that, the plate was incubated at 37°C for 24 h, and the inhibition zones were determined in millimeters using vernier calipers [24].

The susceptibility of MRSA strains to antibiotics was evaluated in vitro, taking into consideration the most common antibiotics used in treatment in the studied area. The antibiotic sensitivity test was performed against some of drugs, such as oxytetracycline (30 μg), gentamicin (10 μg), amikacin (30 μg), tylosin (15 μg), levofloxacin (5 μg), ciprofloxcin (5 μg), moxifloxacin (5 μg), linezolid (25 μg), cefoxitin (30 μg), trimethoprim + sulphamethoxazole (30 μg), and vancomycin (30 μg). The inhibition zones were compared with standard susceptibility zone according to the Clinical and Laboratory Standards Institute (CLSI) [25].

### 2.6. Molecular Diagnosis of *mecA* Gene

Samples of *S. aureus* that had been verified to be resistant to oxacillin and cefoxitin underwent additional processing to confirm the *mecA* gene using PCR. The QIAamp DNA Mini (Qiagen, Hilden, Germany) kit was used for the extraction of DNA of resistant strains. According to Galdiero et al. [26], the extracted DNA was amplified using specific pairs of primers targeting the *mecA* gene, forward primers P1: 5′-TGGCAT TCGTGTCACAATCG-3′ and reverse primer P2: 5′-CTGGAACTT GTTGAGCAGAG-3′, which amplify a product of 310 bp. DNA amplification was performed over 34 cycles, consisting of denaturation at 92 °C for 1 min, annealing at 56 °C for 1 min, and extension at 72 °C for 2 min. A final extension step was conducted at 72 °C for 3 min to complete the reaction. The amplified PCR product was run on a 2% agarose gel, stained with ethidium bromide, and photographed under UV illumination.

### 2.7. Statistical Analysis

The statistical analysis was conducted using SPSS version 24 (SPSS Inc., Chicago, U.S.A.). In a univariate analysis, the risk factors' relationships with the prevalence of SCM were evaluated using the chi-square test. All variables with *p*-value less than 0.25 were subjected to final multivariate logistic regression model. Risk variables were found to have a statistically significant relationship with positive SCM when their *p*-value was less than 0.05. Based on this finding, their odds ratios (ORs) and 95% CI were calculated [27–29].

## 3. Results

The overall prevalence of SCM among examined buffaloes based on CMT was 43.6% (168/385). The prevalence did not differ significantly amongst the examined governorates, with Kafr El Sheikh having the greatest prevalence (46.9%) and Gharbia having the lowest (41.6%), as shown in Table 1.

In the present study, five variables with a *p*-value less than 0.25 in univariate analysis were subjected to a multivariate logistic regression model. The final logistic regression model results showed that the following factors were significantly associated with SCM in water buffaloes: median age (OR = 5.5, 95% CI: 2.8–10.9), parity more than three (OR = 3.1, 95% CI: 1.6–6.1), absence of teat dipping (OR = 7.9, 95% CI: 4.2–14.8), animal with history of previous mastitis (OR = 4, 95% CI: 2.2–7.4), and absence of hand washing between milking (OR = 8, 95% CI: 4.3–14.8) (Table 2).

**Figure 2:**
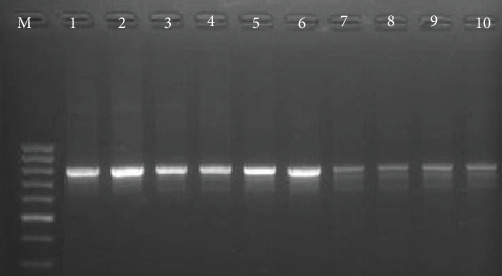
Results of PCR products of *mec*A gene, lane M is a 100 bp DNA ladder, and lane 1 is a positive control. Lane 2–10 are positive samples for MRSA.

Out of 168 *S. aureus* isolates, 104 (61.9%) were resistant to oxacillin and cefoxitin and classified as phenotypic MRSA. The resistant isolates were confirmed using a PCR assay targeting the *mecA* gene, the positive samples gave a detectable band at 300 bp (Figure 2). According to antibiotic sensitivity testing, linezolid demonstrated 100% efficacy, ciprofloxacin 90% efficacy, amikacin and trimethoprim + sulphamethoxazole 80% efficacy, while gentamicin, tylosine, and levofloxacin showed 70%, 65%, and 70% efficacy against MRSA detected in buffalo milk, respectively. In contrast, all MRSA isolates were cefoxitin-resistant (Table 3).

The duration of lactation has no significant effect on the prevalence of SCM in water buffaloes. The study found a significant increase (*p*  < 0.05) in the frequency of SCM in water buffaloes with an age group of >4–8, with a history of mastitis and a parity greater than three. Furthermore, hygienic factors, such as the absence of teat dipping and cleaning of milkers' hands between milking, significantly enhanced the prevalence of SCM (Table 3).

## 4. Discussion

Bovine mastitis has a significant economic impact and the most prevalent disease in dairy bovines and *S. aureus* is the most prevalent cause of mastitis. The development of MRSA in the last years due to misuse of antibiotics in treatment or as a growth promoter has increased the drug-resistant problems and risk to public health.

In the current study, the prevalence of SCM in water buffaloes was 43.6%, which is consistent with Salvador et al. [30] (42.76%). In addition, the prevalence rate is higher than previous reported rates in Philippines (24.22%) [31] and in Bangladesh (10.5%) [32].

According to the PCR assay targeting the *mecA* gene, the prevalence of MRSA is 61.9% (104/168), which is consistent with the findings of Badua et al. [31], who found that the prevalence of MRSA in buffalo milk was 61.54%. Nonetheless, the lower prevalence of MRSA was reported in Pakistan (34%) [33], Germany (16.7%) [34], India (13.1%) [35], Korea (6.3%) [36], and Wisconsin (1.8%) [37].

There are certain discrepancies in the estimation of MRSA prevalence. The prevalence of MRSA could be decreased as a result of overproduction of beta-lactamase or inadequate expression of the *mecA* gene, which results in inconsistent diagnosis [38, 39]. Furthermore, phenotypic expression can be influenced by the pH and osmolality of culture media, and the greater variation found in MRSA strains may contribute to diagnostic challenges [40–45].

All MRSA strains were shown to be 100% sensitive to linezolid but 100% resistant to cefoxitin, which is consistent with earlier findings [29, 33]. Furthermore, the results are consistent with other observations from Egypt, where the majority of *S. aurus* isolates are resistant to cefoxitin and oxytetracycline [9, 46]. Furthermore, it was discovered that none of the MRSA isolates from buffalo milk were cefoxitin sensitive. This finding is in line with the findings of Nemeghaire et al. [47], who reported that all MRSA isolates were cefoxitin resistant.

Cefoxitin and oxacillin are both used as surrogate markers for the detection of MRSA; however, discrepancies between the two can occur. Cefoxitin is considered a better inducer of the *mecA* gene, which encodes the penicillin-binding protein PBP2a responsible for methicillin resistance. As such, cefoxitin disk diffusion and MIC testing are generally more reliable and reproducible compared to oxacillin, especially in borderline or heteroresistant strains [48]. Studies have shown that cefoxitin testing has higher sensitivity and specificity for MRSA detection and is recommended by the CLSI over oxacillin [49]. In contrast, oxacillin may yield false-negative results due to its weaker induction of *mecA*, leading to underestimation of resistance in clinical isolates. Therefore, reliance on oxacillin alone could result in misclassification of MRSA strains, emphasizing the importance of cefoxitin-based testing, particularly in routine surveillance and clinical diagnostics [50].

Biofilms act as a physical barrier, impeding the penetration of antibiotics and host immune responses, thereby enhancing bacterial survival under adverse conditions. Within biofilms, bacteria exhibit altered gene expression, reduced metabolic activity, and increased horizontal gene transfer, all of which contribute to the development and maintenance of methicillin resistance [51]. Additionally, biofilm-associated MRSA infections are more difficult to treat and often result in chronic and recurrent infections [52, 53]. Therefore, understanding the interplay between biofilm formation and antibiotic resistance is essential for the development of effective control strategies.

The results of the present study revealed that the prevalence of SCM increased significantly in buffaloes of median age (4–8 years) with a high number of parities. These results are consistent with those of Badua et al. [31] and Kemal et al. [54], who found that older animals with more calvings had a higher prevalence of SCM. This could be due to pathogenic organisms being more susceptible to udders with relaxed sphincters, and primiparous cows may have more effective defensive mechanisms than multiparous animals [55].

The current findings supported previous studies that found washing the udder before milking can minimize the spread of *S. aureus* in dairy animals [56]. It also demonstrated that teat dipping had a substantial impact on the prevalence of *S. aureus*. It's interesting to note that unclean hands of milkers may be a source of infectious germs spreading during milking procedures; these results are in line with previous research [33]. The presence of *S. aureus* and other infectious bacteria on the udder or teat surface of infected cows is considered as a primary way for transmission of infection between infected and uninfected udder quarters. Therefore, it's clear that the causal organisms could spread quickly from infected to uninfected cows' udders or through the hands of milkers. During lactation, there is virtually little chance of recovery from *S. aureus* infections treated with antibiotics; many infected animals develop chronic infections and must be put to death [56, 57].

Additionally, it can be inferred that animals with a history of mastitis had a higher prevalence of *S. aureus* than animals without a history of mastitis, which is consistent with earlier findings by Mekibib et al. [58] and Selim et al. [44]. The resulting data imply that treating mastitis in animals might not be effective in getting rid of infections, and those infections might be transferred from one lactation to the next [59, 60]. Additionally, it has been reported that buffaloes with three calvings and a previous history of abortion seem to be the most vulnerable to mammary infections since the teat and udder are exposed to harm, and bacteria can readily attach to the teat and enter the gland tissue [56].

## 5. Conclusion

The results of the present study concluded that the high prevalence of SCM and MRSA among water buffaloes in the studied governorates. The prevalence of SCM was significantly associated with age, parity, absence of teat dipping and washing of milker's hand between milking and in animals with a previous history of mastitis. The in vitro antibiotic sensitivity test revealed that the sensitivity of MRSA strains to linezolid and resistance to cefoxitin. Therefore, it is possible to lower the prevalence of MRSA by implementing suitable control measures, conducting routine investigations and treatment protocols, and conducting enough surveillance.

## Figures and Tables

**Table 1:** The prevalence of subclinical mastitis and its association with different variables.

Variable	No. of samples	No. of positive	Percentage of positive	95% CI	Statistic
Locality					*χ* ^2^ = 0.875 *d* = 2 *p* = 0.646
Kafr El Sheikh	130	61	46.9	38.55–55.46	
Gharbia	125	52	41.6	33.34–50.36	
Menofia	130	55	42.3	34.16–50.9	
Age					*χ* ^2^ = 32.409 *d* = 2 *p* < 0.0001*⁣*^*∗*^
2–4	160	48	30.0	23.44–37.5	
>4–8	150	92	61.3	53.35–68.75	
>8	75	28	37.3	27.25–48.64	
Parity					*χ* ^2^ = 31.703 *d* = 2 *p* < 0.0001*⁣*^*∗*^
1	140	40	28.6	21.74–36.55	
2	115	47	40.9	32.32–50.01	
≥3	130	81	62.3	53.74–70.17	
Duration of lactation (months)					*χ* ^2^ = 2.627 *d* = 2 *p* = 0.269
1–3	50	27	54.0	40.4–67.03	
>3–6	120	52	43.3	34.81–52.27	
>6	215	89	41.4	35.02–48.08	
Teat dipping					*χ* ^2^ = 83.486 *d* = 1 *p* < 0.0001*⁣*^*∗*^
Yes	165	28	17.0	12.01–23.43	
No	220	140	63.6	57.1–69.71	
Previous history of mastitis					*χ* ^2^ = 52.677 *d* = 1 *p* < 0.0001*⁣*^*∗*^
Yes	155	33	21.3	15.58–28.39	
No	230	135	58.7	52.24–64.87	
Hygiene of milker's hand during milking					*χ* ^2^ = 84.300 *d* = 1 *p* < 0.0001*⁣*^*∗*^
Yes	168	29	17.3	12.29–23.69	
No	217	139	64.1	57.48–70.15	

Total	385	168	43.6	38.77–48.63	

*⁣* ^*∗*^Results are significant at *p*-Value <0.05.

**Table 2:** Multivariate logistic regression analysis for risk factors related to subclinical mastitis.

Variable	*B*	SE	OR	95% CI for OR		*p*-Value
				Lower	Upper	
Age						
>4–8	1.711	0.347	5.5	2.8	10.9	<0.0001
>8	0.763	0.395	2.1	1.0	4.7	0.033
Parity						
2	0.597	0.365	1.8	0.9	3.7	0.012
≥3	1.126	0.344	3.1	1.6	6.1	0.001
Teat dipping						
No	2.066	0.321	7.9	4.2	14.8	<0.0001
Previous history of mastitis						
No	1.398	0.308	4.0	2.2	7.4	<0.0001
Hygiene of milker's hand during milking						
No	2.076	0.314	8.0	4.3	14.8	<0.0001

*Note: B*, logistic regression coefficient

Abbreviations: CI, confidence interval; OR, odds ratio; SE, standard error.

**Table 3:** Antimicrobial sensitivity test results against methicillin-resistant *Staphylococcus aureus*.

Antibiotic disc	Potency	S	I	R
Oxytetracycline	30 μg	35	25	40
Gentamicin	10 μg	70	0	30
Amikacin	30 μg	80	20	—
Tylosin	15 μg	65	20	15
Levofloxacin	5 μg	70	15	15
Ciprofloxcin	5 μg	90	—	10
Linezolid	25 μg	100	—	—
Trimethoprim + sulphamethoxazole	30 μg	80	—	20
Cefoxitin	30 μg	—	—	100

## Data Availability

The datasets used and/or analyzed during the current study are available from the corresponding author upon reasonable request.
